# Identification of a Novel β-Defensin Gene in Gilthead Seabream (*Sparus aurata*)

**DOI:** 10.1007/s10126-024-10367-z

**Published:** 2024-09-11

**Authors:** M. Ferez-Puche, Jhon A. Serna-Duque, Alberto Cuesta, Álvaro Sánchez-Ferrer, María Ángeles Esteban

**Affiliations:** 1 Immunobiology for Aquaculture Group, Department of Cell Biology and Histology, Faculty of Biology, University of Murcia, 30100 Murcia, Spain; 2 Department of Biochemistry and Molecular Biology, Faculty of Biology, University of Murcia, 30100 Murcia, Spain

**Keywords:** Beta-defensin, Antimicrobial peptides, Gene duplication, Evolution, Teleosts

## Abstract

The excessive use of antibiotics in aquaculture favors the natural selection of multidrug-resistant bacteria, and antimicrobial peptides (AMPs) could be a promising alternative to this problem. The most studied AMPs in teleost fish are piscidins, hepcidins, and β-defensins. In this work, we have found a new gene (*defb2*) encoding a type 2 β-defensin in the genome of gilthead seabream, a species chosen for its economic interest in aquaculture. Its open reading frame (192 bp) encodes a protein (71 amino acids) that undergoes proteolytic cleavage to obtain the functional mature peptide (42 amino acids). The genetic structure in three exons and two introns and the six characteristic cysteines are conserved as the main signature of this protein family. In the evolutionary analysis, synteny shows a preservation of chromosomal localization and the phylogenetic tree constructed exposes the differences between both types of β-defensin as well as the similarities between seabream and European seabass. In relation to its basal expression, β-defensin 2 is mostly expressed in the intestine, thymus, skin, and gonads of the gilthead seabream (*Sparus aurata*). In head kidney leucoytes (HKLs), the expression was very low and did not change significantly when stimulated with various immunocompetent agents. However, the expression was significantly down-regulated in the liver, head–kidney, and blood 4 h post-injection with the fish pathogen *Vibrio harveyi*. When infected with nodavirus, the expression was downregulated in brain at 7 days post-infection. These results denote a possible complementarity between the expression patterns of β-defensins and hepcidins. Further studies are needed to analyze gene duplications and expression patterns of β-defensins and describe their mechanism of action in seabream and other teleost fish.

**Supplementary Information:**

The online version contains supplementary material available at 10.1007/s10126-024-10367-z.

## Introduction

The use of antibiotics in aquaculture to prevent diseases and maintain water quality exerts a selective pressure that favors the natural selection of multi-resistant bacteria. The exponential growth of antibiotic resistance in recent decades has promoted new research to find alternatives to reduce the use of antibiotics in human medicine and veterinary science. In this regard, antimicrobial peptides (AMPs) could be a promising option (Lai and Gallo 2009; González et al. 2021). AMPs are small peptides (20–50 aa) with a wide range of biochemical properties; including cationic, anionic, and neutral molecules that play an essential role in the innate immune response (Wang 2023). They are classified into three groups based on their structure (alpha helix conformation, cyclic, and semicyclic conformation) and its amphipathic properties due to the combination of alpha helix and beta strands, essential to their antimicrobial activity (Dias and Franco 2015). Widely distributed from invertebrates to mammals, AMPs are primarily expressed in skin and mucosal epithelia, where they prevent colonization by pathogens (Yang et al. 2002; Bulet et al. 2004). Moreover, they participate in other functions such as endotoxin neutralization, leucoyte chemotaxis, immunomodulation, angiogenesis, or iron metabolism (Bulet et al. 2004; Guaní-Guerra et al. 2010; Cuesta et al. 2011).

In teleost fish, the importance of the innate immune response is greater than in terrestrial vertebrates, since aquatic ecosystems contain a higher number of pathogens to which fish are continuously exposed (Katzenback 2015). In addition, the adaptive immune response is less developed (Nam et al. 2010). In these animals, AMPs tend to organize into gene clusters and are expressed as a pro-peptide that undergoes proteolytic cleavage, releasing the mature peptide (typically between 20 and 30 amino acids). The most studied AMPs in teleost fish are piscidins, hepcidins, and defensins (Mookherjee et al. 2020). Defensins are small (3.5–4.5 kDa), cationic, and amphipathic peptides with six conserved cysteine residues. They are also distributed from invertebrates to vertebrates and can be classified as α-, β- or θ-defensins depending on the position of the cysteines and the topology of the disulfide bonds (Das et al. 2022). The α and β-defensins have a structure composed of β strands, while the θ-defensins are structured in loops*.* The β-defensins exhibit a genomic organization in two exons separated by an intron, so the six conserved cysteines are in exon 2 (Zou et al. 2007). However, in teleost fish, β-defensins follow a genomic organization of three exons and two introns, with the six conserved cysteines distributed between exon 2 (4 cysteines) and exon 3 (2 cysteines) (Nam et al. 2010).

Different β-defensins have been studied and characterized in a wide range of teleost fish but very few of them are of interest in aquaculture. In zebrafish (*Danio rerio*), one of the most common model organisms, three β-defensins have been characterized: BD-1, BD-2, and BD-3. BD-1 and BD-3 are expressed in gills, gonads, intestine, liver, head-kidney, skin, and spleen of healthy fish, while BD-2 is mildly expressed in the intestine (Zou et al. 2007). In medaka fish (*Oryzas latipes*), a model organism of marine fish, β-defensins are mainly expressed in the liver and head-kidney, with specific antimicrobial activity against gram negative bacteria (Zhao et al. 2009). In rainbow trout (*Oncorhynchus mykiss*), four β-defensins have been described, expressed in intestine, head-kidney, and gills, and two of them (BD-2 and BD-3) are overexpressed after infection with bacteria (Casadei et al. 2009). In European seabass (*Dicentrarchus labrax*), a species of great interest in aquaculture, two β-defensins with low sequence identity have been found, both conserving the six characteristic cysteines (Barroso et al. 2021). Finally, in a previous work performed in our laboratory, a β-defensin (saBD) was described in gilthead seabream (*Sparus aurata*), and it was designated as β-defensin 1 (*defb1* gene) (Cuesta et al. 2011). It has constitutive expression in several tissues (skin, head-kidney, and gills) and antimicrobial activity against gram-positive (*Bacillus subtilis*) and gram-negative (*Vibrio anguillarum*) bacteria. It is also expressed in vitro in head-kidney leucocytes, where expression is higher after stimulation with oligodeoxynucleotides (ODNs) and the fish pathogen *V. anguillarum* (Cuesta et al. 2011). The presence of two or more β-defensins in teleost fish could have its origin in the whole genome duplications these species have undergone during its evolution (Meyer and Van de Peer 2005; Serna-Duque et al. 2022). The aim of this paper is to look for more β-defensin genes and analyze their expression patterns, characteristics, and possible antimicrobial properties of the functional peptides they encode. Our hypothesis of the existence of more β-defensin coding genes in the genome of gilthead seabream is based on the similarity of this species with European seabass, where two β-defensins have already been found (Barroso et al. 2021) and also on its evolutionary relation with the rest of teleost fish previously mentioned. We have identified a new gene (*defb2*) encoding a type 2 β-defensin whose genomic organization and synteny has been studied and its expression patterns evaluated by RT-PCR and real-time PCR, as well as an in silico functional characterization of the defensin protein it encodes and its mature peptide.

## Materials and Methods

### Gene Identification and Gene-mRNA-Protein Sequence Comparative Analysis

The assembled reference genome of gilthead seabream, *Sparus aurata* (GCA_900880675.1 fSpaAur1.1, NCBI *Genome*), was used for all bioinformatic analyses. As defensin-like protein sequences were not found in the genome-derived protein database (*NCBI S. aurata* Annotation Release 100), we opted to search for these genes using Genomicus 104.02. The β-defensin 2 (MZ198754) from seabass was used for BLASTp in Genomicus and allowed us to find an orthologous gene in gilthead seabream genome, *defb2* gene (ENSSAUG00010026539, Ensembl (Barroso et al. 2021). The gene information and structure were retrieved from Ensembl *Gene*. The evolutionary conservation and protein family by BLASTp and Pfam were verified. The previously identified seabream β-defensin (*defb1*, FM158209) and new *defb2* sequences were compared from coding (CDS) and protein sequences by global pairwise alignment (EMBOSS Needle, EMBL-EBI) and visualized using ESPript (Gouet et al. 1999) (Table 1).

**Table 1:** Accession numbers of *defb* genes, mRNAs, proteins, and primers in gilthead seabream (Ensembl, NCBI)

β-Defensin name		Gene		mRNA	Protein	Primer
*defb1*	ENSSAUG00010014090		ENSSAUT00010034981 (FM158209, NCBI)		ENSSAUP00010033200	F-CCCCAGTCTGAGTGGAGTGT R-AATGAGACACGCAGCACAAG
*defb2*	ENSSAUG00010026539		ENSSAUT00010069764		ENSSAUP00010066624	F-TTCTCCTGATGCTCGCAGTC R-ACCGTGATGACCAACGATGT

### Genomic Synteny and Phylogenetic Analysis

Genomic synteny was performed in Genomicus 104.02 by BLASTp and visualized by AlignView (https://www.genomicus.bio.ens.psl.eu/). This used 129 fish genomes from a common ancestor Actinopterygii (~ 386 Mya) and low-coverage genomes were removed; these assembly genomes can be found in *Ensembl*. Phylogenetic tree was inferred using the Neighbour-Joining method by MEGA X with 10,000 replicates in the bootstrap test. The evolutionary distances were computed using the p-distance method and are in the units of the number of amino acid differences per site. This analysis involved 25 amino acid sequences including gilthead seabream β-defensin proteins (Table 2). All ambiguous positions were removed for each sequence pair (pairwise deletion option). Hepcidin 1 from gilthead seabream, a protein belonging to a different family of AMPs, was used as an outgroup to check that the alignment has been carried out correctly.

**Table 2:** Accession numbers of β-defensin proteins used in phylogenetic tree (Uniprot)

β-Defensin name	Species	Accession number
β-Defensin 1	*Sparus aurata*	A0A671W2Y0
β-Defensin 2	*Sparus aurata*	A0A671YY13
β-Defensin 1	*Dicentrarchus labrax*	A0A7R6RH83
β-Defensin 2	*Dicentrarchus labrax*	A0A8C4DI51
β-Defensin 1	*Paralichthys olivaceus*	D2WPC8
β-Defensin 2	*Paralichthys olivaceus*	D2WPC9
β-Defensin 1	*Danio rerio*	A7E2H0
β-Defensin 2	*Danio rerio*	I3ISS9
β-Defensin 3	*Danio rerio*	A1IVN1
β-Defensin	Oreochromis niloticus	U3PYC5
β-Defensina 1	*Oncorhynchus mykiss*	Q14QQ6
β-Defensina 2	*Oncorhynchus mykiss*	C9WX69
β-Defensina 1	*Takifugu rubripes*	D5A7I4
β-Defensina	*Epinephelus cocoides*	Q7T0L3
β-Defensina 1	*Salmo salar*	A0A3G9EPZ0
β-Defensina 2	*Salmo salar*	A0A3G9ECV7
β-Defensina	*Argyrosomus regius*	A0A286RTJ3
β-Defensina	*Ranitomeya imitator*	A0A821WRU0
β-Defensina	*Acipenser dabryanus*	A0A481WHH5
β-Defensina 1	*Sus scrofa*	O62697
β-Defensina 1	*Mus musculus*	P56386
β-Defensina 2	*Mus musculus*	P82020
β-Defensin 1	*Homo sapiens*	P60022
Hepcidin 1	*Sparus aurata*	B2RFG6

### Animals, Organs, and Cells

Specimens of the seawater teleost gilthead seabream (300–500 g body weight) were acquired from a local farm (Murcia, Spain) and transferred to the University of Murcia aquaria. Fish were kept in 450–500 L running seawater (28 ‰ salinity) aquaria at 24 ± 2 °C and with a 12-h light:12-h dark photoperiod. Animals were fed daily with 1% body weight on a commercial pellet diet (Skretting) and acclimatized for 15 days prior to the experiments. All animal studies were carried out in accordance with pertinent bioethical regulations (Esteban et al. 2013). Fragments of the brain, skin, gonad, liver, gut, gill, head-kidney (HK), spleen, thymus, and samples of blood were obtained and immediately frozen in TRIzol Reagent (Invitrogen) for later RNA isolation. The HK leucocytes (HKL) were isolated and maintained in supplemented Leibovitz’s L15 medium (Gibco) at 25 °C (Esteban et al. 2013). Cell viability was determined by the trypan blue exclusion test. Nodavirus (VNNV) (strain 411/96, genotype RGNNV) were propagated in the SSN-1 cell line at 25 °C (Frerichs et al. 1996). Virus stocks were titrated in 96-well plates according to previous experiments (Chaves-Pozo et al. 2012).

Pathogenic bacteria *V. harveyi* (ATCC 35084) was grown in tryptic soy broth (Laboratorios Conda) supplemented with 1.5% NaCl for 24 h at 22 °C (Cámara-Ruiz et al. 2021; Serna-Duque et al. 2023). Bacterial cell cultures were adjusted to 10^8^ CFU mL^−1^ (0.4 Abs) using absorbance at 600 nm. For heat-killing, bacteria was incubated at 60 °C for 30 min (Cuesta et al. 2011).

### In Vitro* Head-Kidney Leucocyte Stimulation*

The HKLs from five different fish were incubated with different stimuli. HKLs (10^7^ cells mL^−1^) were incubated in flat-bottomed 48-well microtiter plates (Nunc) with: culture medium alone (control), lipopolysaccharide (5 µg mL^−1^; Sigma), poly I:C (25 µg mL^−1^; Sigma), synthetic unmethylated cytosine-phosphodiester-guanosine oligodeoxynucleotide 1668 (CpG ODN 1668; sequence TCCATGACGTTCCTGATGCT; 50 µg/mL; Eurogentec), heat-inactivated *Vibrio harveyi* (10^8^ CFU mL^−1^), or nodavirus (10^6^ TCID_50_ mL^−1^) (Cuesta et al. 2011). After 24 h of incubation at 25 °C, HKLs were washed and stored in TRIzol Reagent at − 80 °C for later isolation of RNA.

### In Vivo Stimulation of Seabream Defensin Transcription by *Bacteria*

Seabream specimens were intraperitoneally injected with 1 mL of sterile PBS alone (control) or containing 10^8^ CFU mL^−1^ (0.4 Abs) of *V. harveyi*. The liver, head-kidney (HK), skin, anterior intestine, and spleen were collected after 4 h post-injection and processed to obtain the cDNA. Untreated fish were also sampled.

### In Vivo* Stimulation of Seabream Defensin Transcription by Virus*

Specimens of gilthead seabream received an intramuscular injection of culture medium containing 10^6^ NNV TCID_50_ (Chaves-Pozo et al. 2012). HK and brain organs were sampled 1, 7 days after the viral injection and were stored for RNA isolation.

### RT-PCR and Real-Time PCR

Total RNA was isolated from TRIzol Reagent frozen samples following the manufacturer’s instructions. The quality of RNA was analyzed using a Nanodrop ND-2000 (Nanodrop Technologies) and Agilent 2100 Bioanalyzer System (Agilent Technologies) (Supplementary Fig. 1). One microgram of total RNA was treated with DNase I to remove genomic DNA, and the first strand of cDNA was synthesized by reverse transcription using the ThermoScript™ RNAse H − Reverse Transcriptase (Invitrogen) with an oligo-dT12–18 primer (Invitrogen). Primer design was performed by Oligo Perfect software tool (Invitrogen), and in silico specificity of all possible predicted primers was verified by Primer-BLAST using the seabream whole genome (Table 1). Fast qPCR was performed with a QuantStudio™ 5 real-time PCR as previously indicated, with the identified specific primers and cDNA samples. Reaction mixtures were incubated for 10 min at 95 °C, followed by 40 cycles of 15 s at 95 °C, 1 min at 60 °C, and finally, 15 s at 95 °C, 1 min 60 °C, and 15 s at 95 °C. For experimental verification of primer specificity, qPCR products from the most concentrated cDNA samples were used for high-resolution melt analysis (QuantStudio™ 5 Flex) and for Sanger sequencing (3500 Genetic Analyzer). Melt curves from each amplicon were compared to predicted curves by uMelt^SM^, and the predicted amplicon sequences were compared with the Sanger sequenced products by pairwise alignment. For gene expression in tissues and HKLs, Ct values from defensin mRNAs were normalized to *ef1a* and ribosomal protein S18 (*s18*) housekeeping genes (Livak and Schmittgen 2001).

### *Functional Characterization of Defensin Proteins and Mature Peptides *In Silico

SignalP 6.0 was used to predict the signal peptide and mature peptide in gilthead seabream defensin proteins (Table 1). Physicochemical properties of two mature defensins were studied by *Jalview* 2.11 and *ProtParam* and APD3. Antimicrobial activities for mature peptides were performed in CAMPR3 and ClassAMP. Structure prediction of the two defensin sequences used in this work was performed using AlphaFold2 (Jumper et al. 2021). The model with the highest confidence score was used for subsequent analyses. Structural alignment and figures were performed with PyMol (https://pymol.org/2/).

### Statistical Analysis

Data in figures are represented as mean ± SEM, statistical differences between control and stimulated HKLs were analyzed by one-way ANOVA (Prism 9), while statistical differences of in vivo defensin expression in stimulated seabreams were analyzed by the Student *t*-test (Prism 9).

## Results

### Identification of Novel Duplicated β-Defensin Gene in the Genome of Gilthead Seabream

**Fig. 1:**
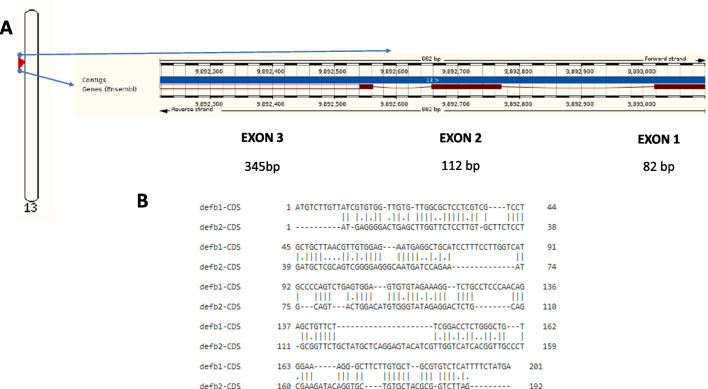
**A** Genomic location and gene structure of gilthead seabream *defb2* gene in chromosome 13 (9892–9893 kbp). **B** Pairwise alignment between defensin genes from seabream at nucleotide level, compared from coding sequences (CDS)

**Fig. 2:**
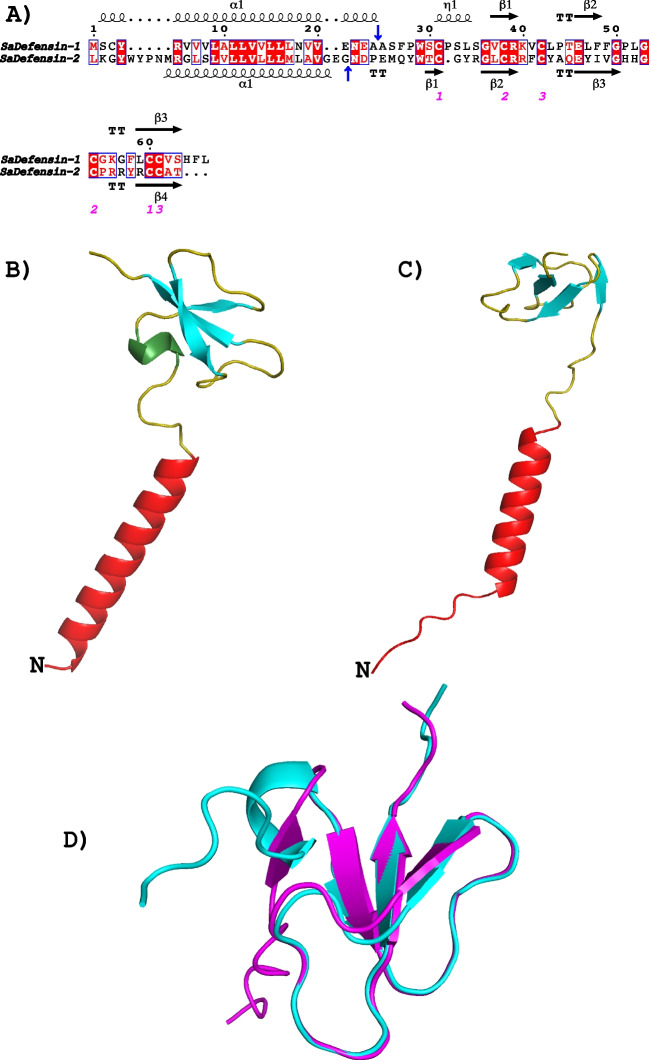
Sequences and structures of the two gilthead seabream defensins. **A** Alignment of the two sequences. Strictly conserved residues have a red background. Symbols above or below the sequences represent secondary structure, springs represent helices, and arrows represent β-strands. Blue arrows represent signal peptide cleavage points. Numbers in magenta represent disulfide bridges. **B** Structural model of Defensin 1. Signal peptide in red, helix 3_10 in green, and β-strands in cyan. **C** Structural model of Defensin 2. The colors are the same as in **B**. **D** Structural alignment of the mature sequences of the two defensins. Defensin 1 in cyan, Defensin 2 in magenta

The gene encoding β-defensin 2 (*defb2*) of gilthead seabream is located in a 9892–9893 kbp region of chromosome 13 (ENSSAUG0001010026539) (Table 1) and has a total length of 882 bp (Table 3). This gene is composed of three exons and two introns (Fig. 1A, Table 3). In fact, the total length of the exons is 539 bp, the length of exon 1 being 82 bp, that of exon 2, 112 bp and that of exon 3, 345 bp. The length of intron 1 (1–2 exons) is 248 bp and the length of intron 2 (2–3 exons) is 95 bp. Within the coding sequence of the *defb2* gene, the largest open reading frame (ORF) is 192 bp and encodes a protein of 71 amino acids (Table 3). Comparison of defensin sequences shows low identity at the transcript level (46.2%) (Fig. 1B) and at the protein level (30.9%), whereas protein similarity barely reached 45.6% (Fig. 2A).

**Table 3:** Comparison of genes encoding β-defensin 1 and 2 proteins in gilthead seabream (Ensembl)

Parameter	*defb1*	*defb2*
Length of the complete gene*	665	882
Length of the coding sequence (exons)*	445	539
Number of exons	3	3
Length exon 1*	84	82
Exon 2 length*	121	112
Exon 3 length*	240	345
Number of introns	2	2
Intron length 1/2*	127	248
Intron length 2/3*	93	95
ORF length*	201	192
Protein length **	66	71
Signal peptide length**	24	28
Mature peptide length**	42	43

^*^Nucleotides; **amino acids

β-Defensin 2 is synthesized as a pro-peptide. Its signal peptide, characterized by a long a-helix, is eliminated by cleavage at GND^**↓**^ND) (Fig. 2A). Moreover, the mature peptide presents only β-strands, namely, 4 (Fig. 2B, C). The six characteristic cysteines allow the formation of 3 disulfide bridges, with a 123–213 pattern (Fig. 2A) which allows that when a structural alignment of the mature peptides is made, the 3 β-strands and the β-turns align perfectly. As for their physicochemical properties (Table 4), BD2 presents a positive charge (+ 4), hydrophobicity (4.4), and a positive Boman index (2.94 kcal/mol).

**Table 4:** Physicochemical and antimicrobial properties of defensin mature peptides from gilthead seabream

Defensin protein	Def1	Def2
Length (aa)	42	43
MW	4495.429	5207.98
pI	8.35	8.90
Net charge	+ 2.25	+ 4.5
Ratio of hydrophobic residues (%)	50	33
GRAVY	0.79	− 0.87
W-W hydrophobicity	− 4.3	4.4
Boman index (kcal/mol)	− 0.38	2.94
AMP PROBABILITY*	0.863	0.735
Antimicrobial activity**	Antibacterial 0.99	Antifugal 0.96

*GRAVY*, Grand average hydropathy value of the peptide; *W-W hydrophobicity*, Wimley-White whole-residue hydrophobicity of the peptide. *Boman index*, protein-binding potential. *Results with support vector machine (SVM) classifier from CAMPR3. **Results with support vector machines classifier from Class AMP

### Syntenic and Phylogenetic Analysis of β-Defensin 2

**Fig. 3:**
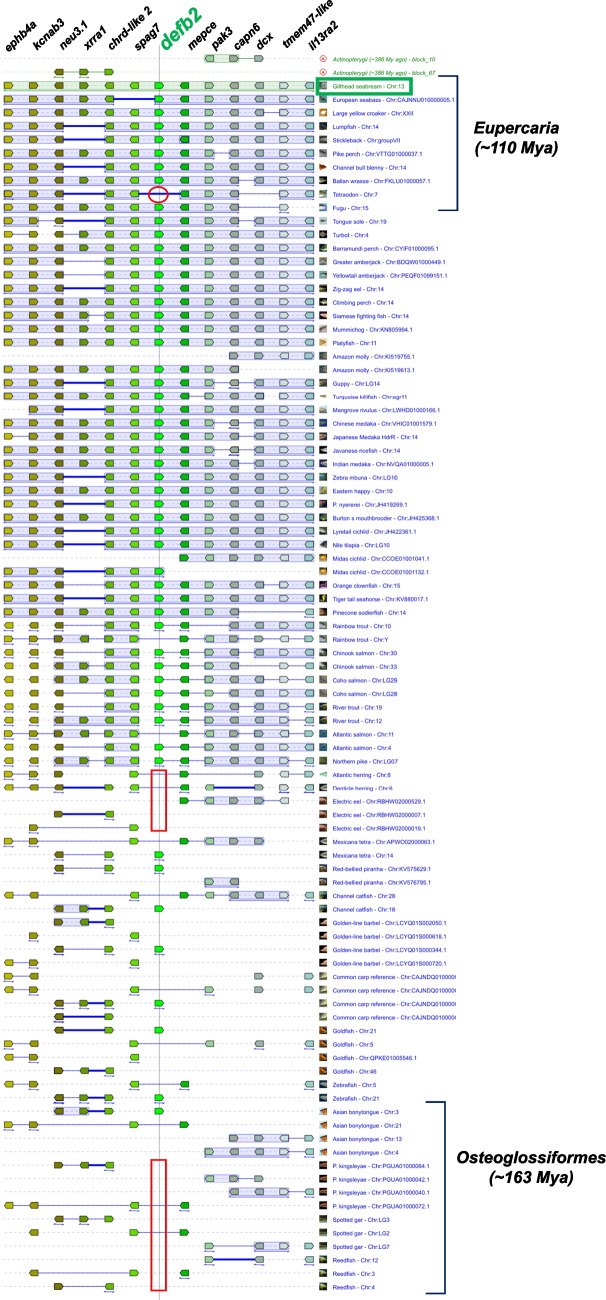
Genomic synteny of *defb2* gene of teleost fish from *Actinopterygii* ancestor in 9892–9893 kbp region of gilthead seabream chromosome 13. Pentagons represent the genes and the direction of the strand. The syntenic conserved orthologs or gene blocks are shown in matching colors. A thin line between two genes is equivalent to a break in the continuity of the alignment. A thick blue line between two genes is equivalent to a “gap” in the alignment of the extant species. A thin double-headed arrow under a block of genes indicates that the order of the genes shown was reversed. Syntenic gap is indicated by a red square or circle, and the studied fish species, *S. aurata*, is highlighted by green square

Syntenic analysis shows that the *defb2* gene is located among other genes encoding membrane proteins and proteins involved in defense against pathogens, which makes sense considering that defensins are AMPs. The *defb2* gene is flanked by the *spag7* gene to its left and *mepce* to its right on gilthead seabream chromosome 13. Most of these flanking genes encode proteins integrated into the plasma membrane, such as Ephb4a or Kcnab3, or are involved in defense and protection against external damage, such as Il13ra2 or *X*rra1 (Fig. 3).

**Fig. 4:**
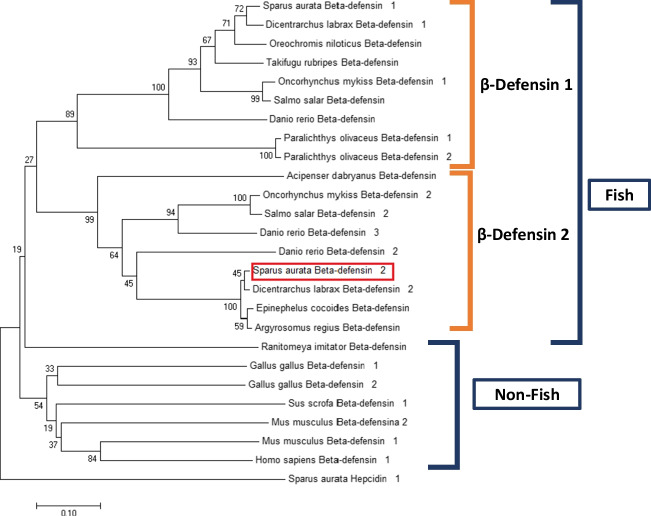
Phylogenetic tree performed with Neighbor-Joining method by MEGA X of 24 fish defensin proteins, including those identified from gilthead seabream and accession numbers for amino acid sequences. The tree was drawn to scale, with branch lengths in the same units as those of the evolutionary distances used to infer the phylogenetic tree. The percentage of replicate trees in which the associated taxa clustered together in the bootstrap test (10,000 replicates) are shown next to the branches. The clades are indicated in phylogenetic tree. The novel seabream sequence is surrounded by red square. Outgroup is represented by hepcidin protein from *S. aurata*

The phylogenetic tree resulting from the alignment of the selected proteins shows that gilthead seabream’s β-defensin 2 is mainly related to its orthologue from sea bass, with which it shares a common ancestor (internal node), followed by β-defensin 2 from grouper and croaker fish (Fig. 4).

### Tissue Expression of β-Defensin 2

**Fig. 5:**
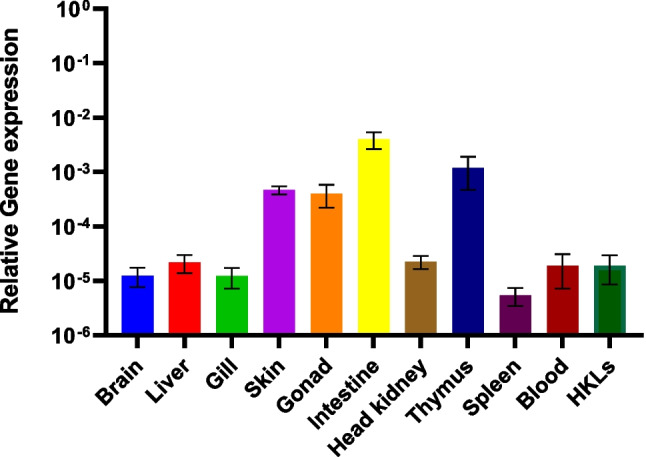
β-Defensin 2 basal expression studied by real-time PCR in different tissues in naïve gilthead seabreams. Data are shown as the mean gene expression relative to the expression of endogenous control *ef1α* and ribosomal protein S18 genes ± SEM (*n* = 3). HKLs, head kidney leucocytes

The gene expression of *defb2* was studied in ten different tissues/organs from non-stimulated seabream specimens. Overall, the *defb2* gene presents a low relative basal expression in all analyzed organs compared to housekeeping genes (Fig. 5). The organs where expression is highest are the intestine and thymus, followed by the skin and gonad. In contrast, expression is notably lower in the remaining organs, with the spleen showing the lowest level of gene expression. Also, it is worth mentioning the similar amounts of mRNA in both blood and HKLs.

### Expression of β-Defensin 2 in Stimulated HKLs

**Fig. 6:**
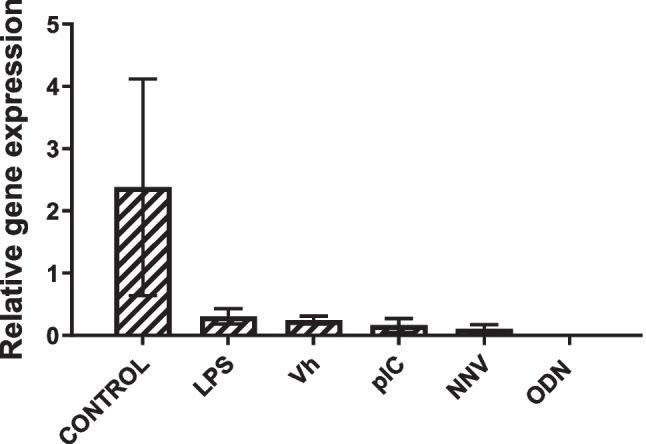
Gene expression of *defb2* in stimulated gilthead seabream head kidney leucocytes (HKLs) in vitro. Gilthead seabream HKLs were incubated for 24 h with culture medium alone (Control), lipopolysaccharide (LPS), poly I:C (pIC), CpG ODN (ODN), *V. harveyi* (Vh) or nodavirus (NNV). Results are expressed as the mean ± SEM (*n* = 5). No significant differences (ANOVA; *p < 0.05) with the control were denoted

To evaluate the potential immune roles of the identified *defb2* gene from gilthead seabream, HKLs were incubated for 24 h with some typical stimuli of the immune system. The *defb2* gene shows a very low expression in HKLs, with no significant changes with respect to the control (unstimulated), when these were stimulated (Fig. 6). Generally, a decreasing trend of expression can be observed in stimulated HKLs, in contrast to high variability of control HKLs. Notably, this trend was more pronounced when it cannot be detected expression induced with bacteria-like DNA such as ODNs.

### Expression of β-Defensin Genes After In Vivo Stimulation by Pathogenic *Bacteria*

**Fig. 7:**
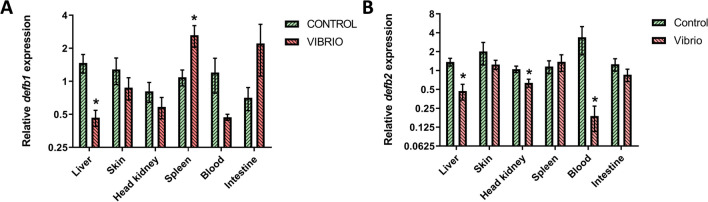
Expression of defensin genes in control and *V. harveyi*-challenged gilthead seabream specimens, in **A** *defb1* and **B** *defb2* gene expression in different tissues at 4 h post-injection. The data are shown as mean ± SEM of expression normalized to endogenous control *ef1a* and *s18* gene. Significant differences (Student *t* test; *p < 0.05) between control and bacteria-challenged samples (*n* = 8) were denoted

Both genes coding for β-defensin 1 and 2 decreases its expression with respect to the control significantly in liver, after 4 h post-injection with *V. harveyi* (Fig. 7). While *defb2* was downregulated in head kidney and even more prominently in the blood, *def1b* was up-regulated in spleen (twofold) at 4 h post injection. In the rest of the organs studied, no significant results were obtained, although there was a tendency toward reduced expression in all of them, except for the spleen and intestine (Fig. 7).

### *Expression of β-Defensin Genes After *In Vivo* Stimulation by Pathogenic Virus*

**Fig. 8:**
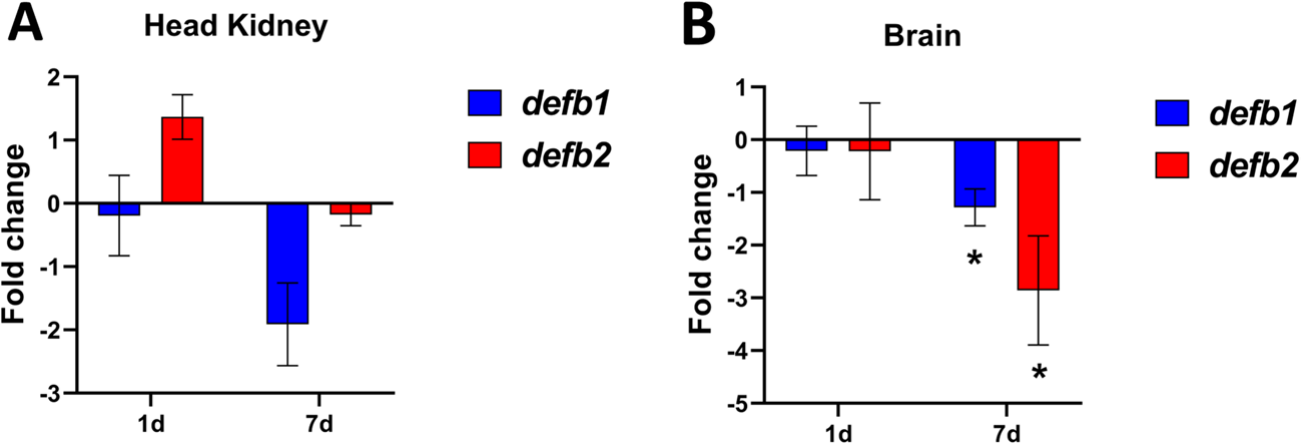
Expression of defensin genes between control and nodavirus-challenged seabream specimens, in **A** head kidney and **B** brain at 1, 7 days post-injection. The data are shown as mean ± SEM of fold-change expression normalized to endogenous control *ef1a* gene. Significant differences (Student *t* test; *p < 0.05) between control and virus-challenged samples (*n* = 4) were denoted

Defensin expression genes on HK and brain on nodavirus infected gilthead seabream was studied at 1- and 7-day post-injection, as viral replication and the anti-viral response are critical at these times. More concretely, at 1-day post-injection, the virus begins to replicate in the brain, and it starts the interferon response. Besides this, at 7-day post-injection, the immune response is very active including interferon response, cell-mediated cytotoxicity, and respiratory burst. Furthermore, transcription of several immuno-relevant genes suggests leucocyte activation and trafficking (Chaves-Pozo et al. 2012). No significant changes in defensin expression were detected in the head kidney (Fig. 8A). Unlike, both *defb* genes were significantly downregulated in brain at 7 days, while there were not changes at first day (Fig. 8B). Likewise, β-defensin expression profiles between HK and brain were different.

## Discussion

### β-Defensin 2 Molecular Characteristics

AMP encoding genes are typically organized in clusters (Pazgier et al. 2006; Dijk et al. 2008; Santana et al. 2021), such as gilthead seabream’s hepcidin genes (Serna-Duque et al. 2022). The hepcidin family has been the most studied in this species and shares several structural and functional characteristics with defensins. Structurally, both are characterized by β-hairpins with disulfide bridges and share a common evolutionary γ-core motif, forming multiple-disulfide scaffolds and serving as cysteine-stabilized host defense peptides (Nigro et al. 2017). Functionally, they seem to exhibit complementarity in expression with defensins.

However, gilthead seabream’s and European seabass’ β-defensin genes are not organized in clusters (Barroso et al. 2021). The discovered defb2 gene of gilthead seabream is genomically organized similarly to the rest of β-defensin encoding genes described in teleost fish (Nam et al. 2010; Cuesta et al. 2011; Harte et al. 2020; Barroso et al. 2021). The nucleotide sequence of *defb2* is longer than that of *defb1* (Table 3) but it does not vary among gilthead seabream’s gene and its ortholog in European seabass. The preservation of the six characteristic cysteines of the β-defensin family is also remarkable in gilthead seabream’s β-defensin 2. These cysteines remain in the mature peptide after proteolytic cleavage due to the importance of the disulfide bonds created between them in the correct protein folding. Therefore, when a structural alignment of the mature peptides is performed, it shows that the 3 β-strands and the β-turns align perfectly, giving a very similar overall structure. Both β-defensin 1 and 2 are synthesized as a pro-peptide, and their signal peptide, characterized by a long a-helix, is eliminated differently: in β-defensin 1 (BD1), the cleavage occurs at the NEA↓AS sequence, whereas in β-defensin 2 (BD2), it occurs at GND↓ND. Moreover, the structure of their mature peptides is also different. While BD1 shows a small 3_10_ helix, followed by 3 antiparallel β-strands, BD2 presents only β-strands, namely, 4. The molecular characterization of *defb2* gene suggests certain functional differences between β-defensin 1 and 2, confirmed with the analysis of their mature peptide properties. The only common characteristic is positive net charge which indicates they are cationic peptides, a characteristic feature of all AMPs. However, β-defensin 1 mature peptide has a high percentage of hydrophobic residues, a positive GRAVY value, and a negative Wimley-White value, indicating high probability to insert in cellular membranes, and a negative Boman index, which manifests a low probable interaction with other proteins. On the contrary, β-defensin 2 mature peptide has a low percentage of hydrophobic residues, a negative GRAVY value, and a positive Wimley-White value, indicating a low probability to insert in cellular membranes, and a positive Boman index, which manifests a probable interaction with other proteins. Based on these in silico results, we suggest that antimicrobial activity of β-defensin 2 could be mediated by protein inhibition via interaction with pathogen proteins instead of by membrane insertion, as predicted for β-defensin 1 in the present study (Kudryashova et al. 2017; Mookherjee et al. 2020).

### Evolutionary Differences of β-Defensin Genes and Proteins

In general, the genomic synteny of *defb2* was highly conserved among the different teleost species of the Actinopterygii ancestor, even in some distant species (Fig. 3). In the Eupercaria block, most genes are conserved; however, gene inversions were found very frequently, and no orthologue of *defb* can be found in the tetraodon fish (*Tetraodon nigroviridis*) due to a syntenic gap, although some fish such as two Clupeiformes (*Clupea harengus*, *Denticeps clupeoides*), electric eel (*Electrophorus electricus*), and most of Osteoglossiformes (*Paramormyrops kingsleyae*, *Lepisosteus oculatus*, and *Erpetoichthys calabaricus*) did not follow the same syntenic block of defensin-like genes due to breaks in genomic alignment.

From another point of view, the phylogenetic tree resulting from the alignment of the selected proteins is divided into two main clades, grouping on the one hand the teleost β-defensins (plus a frog β-defensin) and, on the other hand, those of birds and mammals. Within the β-defensins of teleosts, type 1 (89 bootstrap) and type 2 (99 bootstrap) defensins are differentiated as subclades, with the exception of the olive flounder (*Paralichthys olivaceus*), whose defensins constitute an independent branch within the group of type 1 β-defensins. Similarly, the β-defensin 1 of gilthead seabream was very close to sea bass defensin-1, confirming the division of defensins between evolutionary related-species.

Thus, the presence of several β-defensins in gilthead seabream and other teleost fish may have its origin in the three duplications of its complete genome that this species has undergone during its evolutionary process (Serna-Duque et al. 2022). Another hypothesis is related with the presence of mobile genetic elements, as various retrotransposons are repeated throughout the gilthead seabream genome (Pérez-Sánchez et al. 2019). In gilthead seabream, each gene would have evolved independently, undergoing different modifications in nucleotide and amino acid sequence, which explains the low molecular identity between these two β-defensins. As for the similarity with sturgeon β-defensin, β-defensin type 2 could be the ancestral protein, and type 1 would be an evolved version. Interestingly, the frog β-defensin analyzed most closely resembles the β-defensins of teleost fish, probably due to the evolutionary relationship between fish and amphibians and the preservation of certain genes after the transition from aquatic to terrestrial ecosystems (Bi and Zhang 2021).

### β-Defensin 2 Expression Patterns

The hypothesis of a functional difference between type 1 and 2 β-defensins is supported by the results of defb2 relative expression study. In both gilthead seabream and European sea bass, the basal expression of β-defensin 2 in different organs is higher and more variable than the expression of β-defensin 1, but while β-defensin 2 in gilthead seabream is mainly expressed mostly in the gut, in European seabass, the expression is lower in the gut than in other organs (Barroso et al. 2021). Due to the antimicrobial function of defensins, higher expression is expected in organs exposed to the outside world, such as the skin or gills, and organs of the immune system, such as the thymus and spleen. For this reason, the expression of β-defensin 2 in other organs (intestine, gonads) is of great interest and should be further investigated (Das et al. 2022).

The variations observed in the expression patterns of different organs can be attributed, in part, to the unique functions performed by each organ. Specifically, the liver is responsible for producing acute phase proteins, the main kidney serves as the primary site of hematopoiesis, the blood is where the mature leucocytes are located, and the spleen acts as a secondary lymphoid organ that also facilitates recycling of erythrocytes. However, the different expression patterns of both β-defensins after infection with *V. harveyi* and HKLs stimulation with ODNs and *Vibrio* DNA highlight that further studies are needed to gain a better understanding of the function of AMPs depending on the organ in which they are expressed (Soulliere and Dixon 2017; Ehlting et al. 2021). For other post-infection time intervals, results obtained in other species such as rainbow trout could be used as a basis, in which the expression of β-defensin increases 48 h after infection with *Yersinia ruckeri* (Casadei et al. 2009).

Finally, a possible complementarity between the expression patterns of defensins and hepcidins is suggested: in the first hours post-infection, the liver produces hepcidins and, if the infection is prolonged, metabolism in this organ would switch to producing β-defensins (Nigro et al. 2017; Serna-Duque et al. 2023).

Turning to another aspect of our study, data from nodavirus infection suggest that the two defensins may act at different times, namely, between 48 and 72 h after infection, a period we did not investigate. There is also the possibility that these defensins do not play a role in viral defense. Further research is therefore needed to understand whether and how defensins are involved in the response to viruses, and at what stages of the response. In conclusion, although some interesting discoveries have been made, including the characterization of *defb2* gene and the analysis of the differences between both gilthead seabream’s β-defensins expression patterns, protein folding and mature peptide processing, to confirm all the hypothesis proposed in this paper, it is necessary to continue investigating β-defensins expression in gilthead seabream to clarify their mechanism of action and their coordination with the rest of AMPs in this species.

## Supplementary Information

Below is the link to the electronic supplementary material.

Supplementary file1 (DOCX 131 KB)

## Data Availability

All data generated or analyzed during this study are included in this published article.
