# Supplementary material for: Identification of a Novel β-Defensin Gene in Gilthead Seabream (Sparus aurata)
Source: Mar Biotechnol (NY). 2024 Sep 11;26(6):1219–30. doi: 10.1007/s10126-024-10367-z (PMC11541337; doi:10.1007/s10126-024-10367-z)
Supplement: Supplementary file 1 — Supplementary file1 (DOCX 131 KB) [file 10126_2024_10367_MOESM1_ESM.docx]

**Supplementary File**

**Identification of a novel β-defensin gene in gilthead seabream (*Sparus aurata*)**


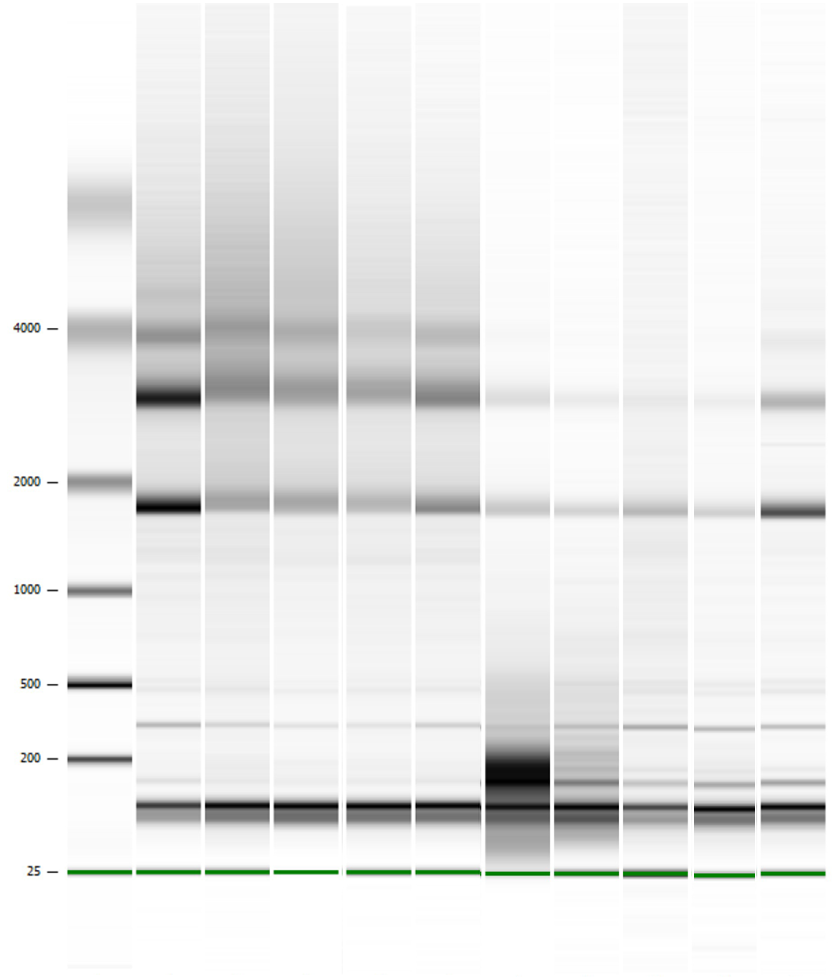


**Fig. S1.** Gel image of RNA samples on the Agilent Technologies 2100 bioanalyzer. Lane 1 contains the ladder (4,000-25 nt). Lanes 2-5 contain liver RNA samples; lanes 6-10 contain spleen RNA samples. Distinct 18S and 28S RNAs bands indicate high-quality RNA.
